# Metabolomic Profile of Oviductal Extracellular Vesicles across the Estrous Cycle in Cattle

**DOI:** 10.3390/ijms20246339

**Published:** 2019-12-16

**Authors:** Julie Gatien, Pascal Mermillod, Guillaume Tsikis, Ophélie Bernardi, Sarah Janati Idrissi, Rustem Uzbekov, Daniel Le Bourhis, Pascal Salvetti, Carmen Almiñana, Marie Saint-Dizier

**Affiliations:** 1 Allice, 37380 Nouzilly, France; julie.gatien@allice.fr (J.G.); sarah.janatiidrissi@allice.fr (S.J.I.); daniel.lebourhis@allice.fr (D.L.B.); pascal.salvetti@allice.fr (P.S.); 2 Institut National de la Recherche Agronomique (INRA), CNRS 7247, University of Tours, IFCE, UMR85 Physiologie de la Reproduction et des Comportements, 37380 Nouzilly, France; pascal.mermillod@inra.fr (P.M.); guillaume.tsikis@inra.fr (G.T.); ophelie.bernardi@inra.fr (O.B.); carmen.alminanabrines@uzh.ch (C.A.); 3 Faculty of Medicine, University of Tours, 37000 Tours, France; rustem.uzbekov@univ-tours.fr; 4 Faculty of Bioengineering and Bioinformatics, Moscow State University, 119991 Moscow, Russia; 5 VetSuisse Faculty, University of Zurich, 8057 Zurich, Switzerland; 6 Faculty of Sciences and Techniques, University of Tours, 37200 Tours, France

**Keywords:** oviduct, fallopian tube, extracellular vesicles, exosomes, metabolomics, energy substrates, amino acids, NMR

## Abstract

Oviductal extracellular vesicles (oEVs) have been proposed as key modulators of gamete/embryo maternal interactions. The aim of this study was to examine the metabolite content of oEVs and its regulation across the estrous cycle in cattle. Oviductal EVs were isolated from bovine oviducts ipsilateral and contralateral to ovulation at four stages of the estrous cycle (post-ovulatory stage, early and late luteal phases, and pre-ovulatory stage). The metabolomic profiling of EVs was performed by proton nuclear magnetic resonance spectroscopy (NMR). NMR identified 22 metabolites in oEVs, among which 15 were quantified. Lactate, myoinositol, and glycine were the most abundant metabolites throughout the estrous cycle. The side relative to ovulation had no effect on the oEVs’ metabolite concentrations. However, levels of glucose-1-phosphate and maltose were greatly affected by the cycle stage, showing up to 100-fold higher levels at the luteal phase than at the peri-ovulatory phases. In contrast, levels of methionine were significantly higher at peri-ovulatory phases than at the late-luteal phase. Quantitative enrichment analyses of oEV-metabolites across the cycle evidenced several significantly regulated metabolic pathways related to sucrose, glucose, and lactose metabolism. This study provides the first metabolomic characterization of oEVs, increasing our understanding of the potential role of oEVs in promoting fertilization and early embryo development.

## 1. Introduction

Exosomes and microvesicles, collectively known as extracellular vesicles (EVs), are membrane-enclosed particles able to transfer a complex selection of molecular compounds from one cell to another, and in this way play key roles in cell-to-cell communications [1]. EVs have been identified in all biological fluids and can be secreted by most cell types. In the female genital tract, EVs have been isolated from follicular, uterine, and oviductal fluids (OF) [2,3,4,5]. EVs from the OF (oEVs), also known as oviductosomes, have gained growing attention in recent years as they may act as natural cargos, bringing key components from the maternal compartment into gametes and embryos for the establishment of pregnancy [5,6,7,8,9]. Oviductal EVs are released by oviductal epithelial cells (OECs), as shown from in vitro studies [5,7], and may originate from the pre-ovulatory follicle at ovulation. Previous studies in mice showed that incubation of spermatozoa with oEVs activates a Ca2+ efflux pomp, PMCA4, which is important for the acquisition of sperm hyperactivated motility and fertilizing competence [6]. In the feline species, oEVs were shown to fuse with the sperm membrane, to improve sperm motility and fertilizing ability, and to prevent premature acrosome reaction in vitro [8]. Recently, we showed that oEVs can reduce polyspermy during in vitro fertilization of pig oocytes without affecting the global fertilization rate [10]. We also demonstrated that the co-incubation of in vitro produced embryos with oEVs increased blastocyst rates, extended embryo survival over time, and improved embryo quality [5]. In mice, oEVs from donor oviduct fluid in the transfer medium were also shown to improve birth rates after the transfer of in vitro produced embryos to recipient mothers [11]. All together, these data support the idea that oEVs play important roles in sperm capacitation, fertilization, and early embryo development.

To further investigate the compounds carried by oEVs potentially playing these roles, we analyzed the proteomic and transcriptomic profiles of oEVs across the estrus cycle revealing proteins, mRNAs, and various types of small non-coding RNAs with important roles in early reproductive events [5,9]. To date, only a few studies have reported the molecular content of oEVs (reviewed in [12]). In the present study, we focused on the metabolomic profile of oEVs, the least studied component of EVs in all research areas, with still no available data on oEVs. If we consider that metabolites represent an intermediate or end point of any cellular process, they can reflect the phenotype printout of a cell at a specific moment. Thus, monitoring metabolic changes in any biological fluid under a variety of physiological or pathological conditions may be seen as the goldmine of biomarkers of health condition [13]. Moreover, determining the metabolite composition of EVs can reveal important aspects in cell-to-cell communication.

On the other hand, it is widely accepted that the ovarian steroid hormones progesterone (P4) and estradiol-17β (E2) regulate all components of the oviduct secretory activity [14] including oEV content [9] and also influence the metabolic content of the OF [15]. Moreover, in mono-ovular species like the bovine and human, the side of ovulation may also affect the molecular composition of the proximal OF by topical hormonal regulations and putative input of the ovulatory follicle. Thus, the objectives of the present study were to analyze the metabolomic profile of oEVs and examine whether the oEV metabolite content is under the hormonal control of the estrous cycle and is dependent on the side relative to ovulation.

## 2. Results

### 2.1. Characterization of Oviductal Extracellular Vesicles Used for Metabolomic Profiling

The oEVs samples collected from cyclic cows and used for NMR analysis are shown in Table 1.

**Figure 1:**
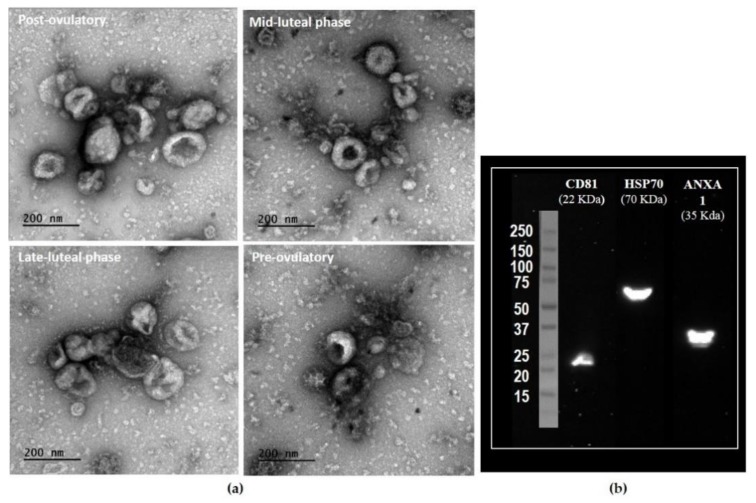
Characterization of bovine oviductal extracellular vesicles (oEVs). Representative images of exosomes (30–100 nm) and microvesicles (>100 nm) in oEV preparations observed by transmission electron microscopy (TEM) across the estrus cycle (**a**) and Western blotting characterization of bovine oEVs for known exosomal protein markers (**b**). A pool of samples from four different stages was used, showing that oEVs were positive for CD81, HSP70, and ANXA1.

Transmission electron microscopy (TEM) observation confirmed the presence of oEVs at the four stages of cycle analyzed (Figure 1). Oviductal EVs samples contained a majority of vesicles resembling exosomes (size of 30–100 nm; 76.4 ± 0.8%) and a small population of vesicles resembling microvesicles (>100 up to 500 nm: 23.7 ± 0.8%), with similar proportions among stages, as we previously showed in cattle [9]. Western blotting confirmed the expression of exosomal cytosolic markers (CD81, HSP70, and ANXA1) in the oEV samples (Figure 1).

### 2.2. Metabolite Profile of oEVs across the Estrous Cycle

Proton nuclear magnetic resonance spectroscopy identified 22 metabolites in oEVs (Table 2). The overall oEV metabolite composition showed various classes of metabolites including essential and non-essential amino acids and various energy substrates among other categories (Table 2).

The concentrations of the 15 metabolites could be quantified (Table 3). Among the seven metabolites that could not be quantified, five (glucose, glutamate, leucine, proline, and threonine) were due to a large overlap of their signals with other metabolites and two (formate and ethanolamine) due to signals below the detection threshold. From the 15 metabolites quantified, lactate, myo-inositol, and glycine were the most abundant metabolites throughout the estrous cycle, reaching on average 25–51.5 nmoL mg^−1^ of oEV protein.

### 2.3. Effect of the Stage of the Estrous Cycle and Side of Ovulation on Metabolite Concentrations in oEVs

**Figure 2:**
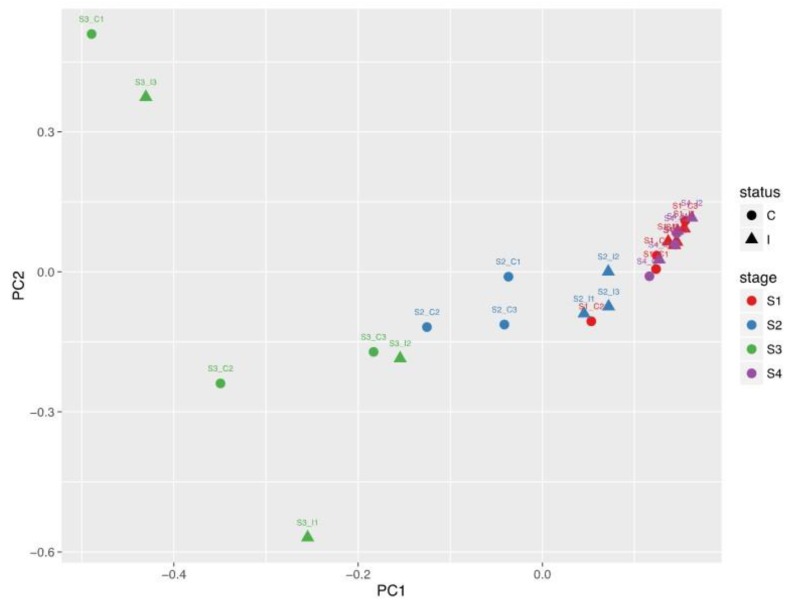
Comparative analysis of the oviduct EV metabolite content across the bovine estrous cycle. Principal component analysis (PCA) of metabolites measured in oEV at four different stages of the estrous cycle. S1, post-ovulation (red); S2, mid-luteal phase (blue); S3, late luteal phase (green) and S4, pre-ovulation (purple) from oviducts ipsilateral (triangles) and contralateral (round spots) to ovulation.

Principal component analysis of the metabolite levels showed a clear separation of metabolite profiles between the peri-ovulatory stages (Post-ov and Pre-ov), which clustered together, and the Mid-lut and Late-lut stages (Figure 2). However, the PCA plot could not discriminate between the two sides relative to ovulation (ipsilateral vs. contralateral).

**Figure 3:**
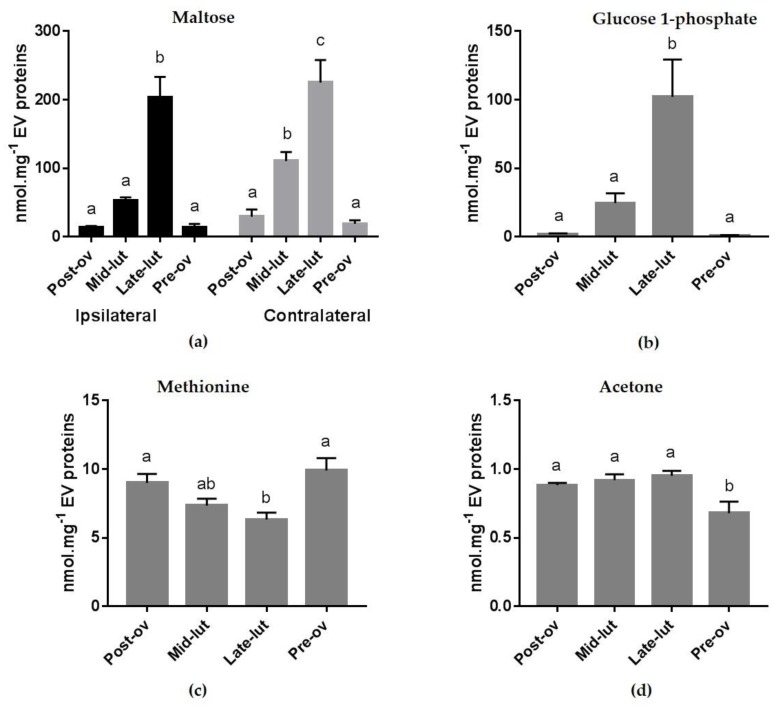
Differential concentrations of specific bovine oEV metabolites across the estrus cycle. Maltose intra-oEV concentrations (nmol.mg−1 of EV protein, (**a**) was affected by the cycle stage and side of ovulation. Glucose-1-P (**b**), methionine (**c**) and acetone (**d**) intra-oEV concentrations were only influenced by the stage of the estrus cycle. For (**b**–**d**), ipsilateral and contralateral concentrations data were pooled as the side of ovulation did not show any significant effect on the metabolite level. (Post-ov: post-ovulatory phase; Mid-lut: mid-luteal phase; Late-lut: late lutal phase; Pre-ov: pre-ovulatory phase).

The ANOVA confirmed the results of the multivariate analysis. Except for maltose, which was slightly affected by the side of ovulation (*p* = 0.04), there was no difference between the ipsilateral and contralateral oviducts or cycle stage × side interaction in the concentrations of any metabolite. The stage of the cycle had a significant effect on the glucose-1-phosphate, maltose, methionine, and acetone concentrations (Figure 3). Glucose-1-phosphate was the metabolite most affected by the cycle stage, showing 100-fold higher levels at the luteal phase than at peri-ovulatory phases (*p* < 0.0001). Similarly, maltose was on average 8- to 14-fold more concentrated at Late-lut than at Post-ov and Pre-ov in both sides relative to ovulation (*p* < 0.0001). In contrast, concentrations of methionine were significantly higher at Pre-ov and Post-ov than at Late-lut (*p* < 0.01). In addition, concentrations of acetone were lower at Pre-ov than at other stages of the cycle (*p* < 0.01).

### 2.4. Pathways Associated with oEVs Metabolites

**Figure 4:**
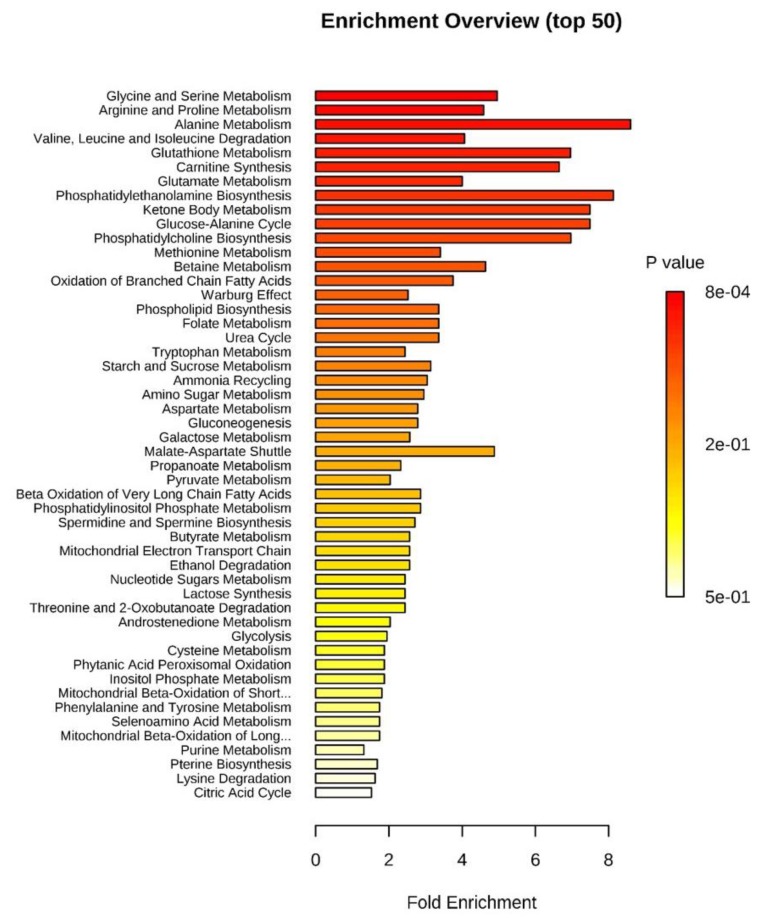
Graphical overview of the over representation analysis (ORA) for all metabolites identified in bovine oEVs generated by MetaboAnalyst 4.0 web-based software. Pathway associated metabolite sets are sorted based on fold enrichment and *p* value. Further details on *p*-values and metabolites included in each pathway are detailed in Table 4.

The over-representation analysis (ORA) of all identified oEVs metabolites against the pathway associated metabolite sets of MetaboAnalyst showed that glycine and serine metabolism was the most significant pathway (*p* < 0.001 and false discovery rate (FDR) < 0.1; Table 4 and Figure 4).

**Figure 5:**
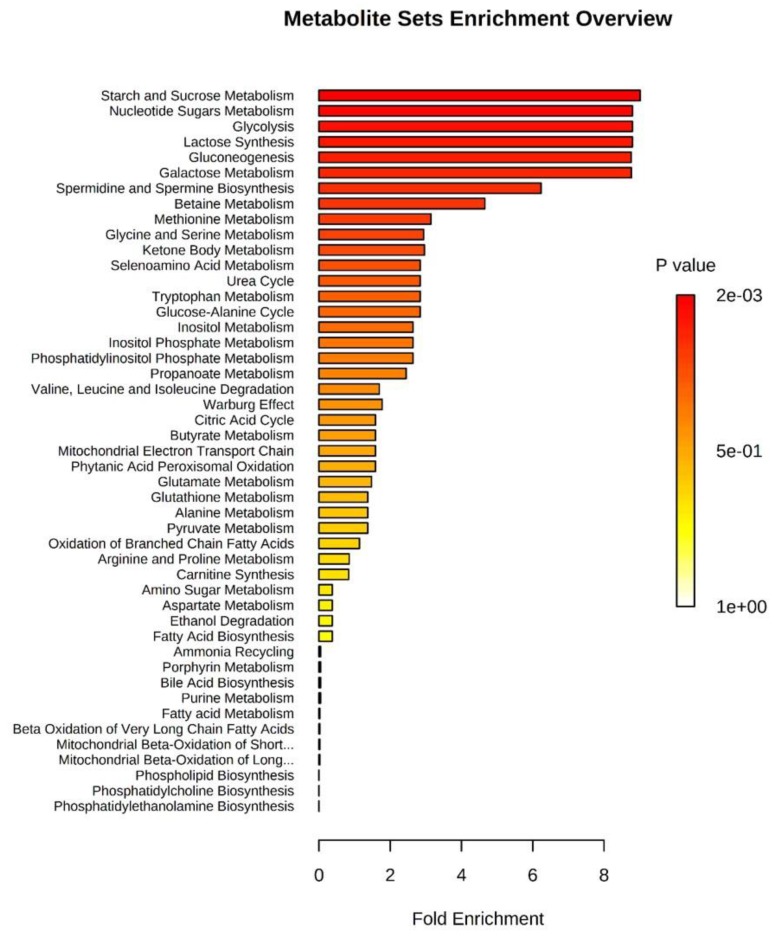
Graphical overview of the quantitative enrichment analysis for all metabolites quantified in bovine oEVs generated by MetaboAnalyst 4.0 web-based software. Pathway associated metabolite sets are sorted based on fold enrichment and p-value after comparison between stages. This bar chart was obtained by comparing Pre-ov vs. Late-lut stages. For each stage, ipsilateral and contralateral data were pooled. Further details on p-values and metabolites included in each pathway are detailed in Table 5.

The quantitative enrichment analysis using the metabolite concentrations at Pre-ov vs. Late-lut stages is shown in Figure 5. Starch (polysaccharide) and sucrose metabolism, nucleotide sugars metabolism, glycolysis, lactose synthesis, gluconeogenesis, galactose metabolism, spermidine/spermine biosynthesis and betaine synthesis were the most regulated pathways between Pre-ov and Late-lut (*p* < 0.01 and FDR < 0.1; Table 5). Similar metabolic pathways related to polysaccharide, sucrose, glucose, and lactose metabolism were significantly regulated by comparing Pre-ov vs. Mid-lut, Post-ov vs. Mid-lut, and Post-ov vs. Late-lut whereas the Post-ov vs. Pre-ov comparison retrieved no significant results.

## 3. Discussion

Currently, there is an increasing number of studies analyzing the metabolomics of EVs in different biological fluids, since both metabolites and EVs are being considered as valuable biomarkers of health conditions and play a crucial part in cell-to-cell communications. In the context of pregnancy, oEVs have been proposed as key modulators of the gametes/embryo-oviduct crosstalk to achieve successful pregnancy [12]. To the best of our knowledge, this study provides the first metabolic characterization of oEVs. Furthermore, we demonstrated that the oEV-metabolite cargo is regulated by the hormonal environment of the estrus cycle, although to a lesser extent than proteins and mRNA in oEVs [5,9].

In the present study, NMR allowed the identification of 22 metabolites in oEVs obtained from cows at four different stages of the estrus cycle. Among them, various classes of metabolites were found such as amino acids, vitamins, energy substrates, and others presented in Table 2. Although the amount of detected metabolites in oEVs was low compared to other metabolomic studies performed by mass spectrometry methods, NMR was selected because of (1) its advantages in highly selective, quantitative, and non-destructive analysis [16], and (2) it was the method used to analyze the metabolite composition of the OF in a previous study performed in our laboratory [15], allowing us a better comparison between data. In fact, most of the metabolites identified here were previously identified and quantified in the bovine OF by NMR [15]. Moreover, the predominant metabolites in oEVs (myoinositol, lactate, and glycine), were also the most abundant metabolites quantified by NMR in the OF by [15]. However, the less abundant metabolites in the OF (i.e., aspartate, choline, mannose, and pyruvate) were not detected in the present study. We can speculate that solute metabolites present in the OF cross the oEV membranes and reach intra-oEV concentrations that mirror those in the OF. Minor metabolites present in the fluid could remain at undetectable levels after being packed into oEVs. Nevertheless, we cannot discard the idea that the methodology used in the present study or the starting oEV volume used limited the detection of those minor metabolites. On the other hand, we identified metabolites such as maltose and acetone, which had not been previously detected in bovine OF [15]. These results suggest that these metabolites may be specifically transported from the tubal fluid into oEVs, reaching higher concentrations inside oEVs than outside. However, the mechanisms by which these metabolites concentrated into oEVs still remains unknown.

In the present study, besides determining whether the hormonal environment of the estrus cycle controls the metabolic profile of oEVs, we also aimed to determine if the side related to ovulation could affect the oEV-metabolite composition, by comparing profiles in ipsilateral versus contralateral oviducts. We thought it was important to include this factor because (1) in previous work from our laboratory, we used pools of ipsilateral and contralateral oviducts [5,9], while others have focused only on the ipsilateral side [17], ignoring the evaluation of the side effect; (2) significant differences in steroid hormones and protein content in the OF were recorded between the two sides relative to ovulation in cattle [18,19]. Moreover, the cow, as a mono-ovular species, is an ideal model to study the effect of the proximity of the ovulatory follicle and post-ovulatory corpus luteum on oviduct physiology. Nevertheless, our results showed that the abundance of metabolites in oEVs was overall not affected by the side relative to ovulation. Only the concentration of maltose was slightly different between the ipsilateral and contralateral oviducts. In the same line, only five metabolites out of the 26 quantified by NMR in the OF were affected by the side of ovulation, with small differences between ovulation sides [15]. Other studies in the field of metabolomics have failed to evidence significant differences in the concentrations of lipids, amino acids, and energy substrates between ipsilateral and contralateral oviducts [20,21,22,23,24]. The contribution of the pre-ovulatory follicle to the content of the OF after ovulation has been under debate for many years [14]. However, the similar metabolite composition between both ovulation sides at Post-ov in oEVs as well as in OF [15], together with other studies above-mentioned does not support this hypothesis.

As spermatozoa and preimplantation embryos are able to uptake oEVs [5,6,8], oEV-metabolites at the pre- and post-ovulatory stages may contribute to their continuous supply of amino acids and energy substrates for the success of early reproductive events. In this sense, we would like to point out that the three most abundant metabolites identified in oEVs (i.e., myoinositol, lactate, and glycine, may have a higher impact on gametes and embryos. Myoinositol and lactate were found to be major energy substrates in oEVs throughout the estrous cycle. Myoinositol is synthesized from glucose and is an energy source for both spermatozoa [25] and embryos [26]. Myoinositol improved bovine sperm motility [27] and embryo development up to the blastocyst stage when added in vitro [28]. Lactate, together with pyruvate, have been identified as the main energy supply for sperm capacitation [29]. After fertilization, the early pre-implantation embryo uses oxidative metabolism to acquire energy, mostly from lactate and pyruvate as the main sources of energy [30]. Glycine, a conditionally essential amino acid, was the third most abundant metabolite in our study and was by far the most abundant amino acid in the oEVs. Using the ORA program of MetaboAnalyst, several amino acid-associated pathways, primarily serine and glycine metabolism, were identified as over-represented in oEVs. Data on the metabolic pathways associated with the establishment of gestation are scarce. However, glycine is known to be important for early embryo development as it is an essential precursor for the synthesis of proteins and nucleic acids [31,32]. Moreover, glycine is required for the rapid proliferation of cells [33]. Glycine also regulates intracellular pH and may have a protecting role against osmotic stress for preimplantation embryos [34].

Among the metabolites identified, glucose-1-P, maltose, methionine, and acetone displayed differential concentrations across the estrus cycle, which may reflect the requirements of gamete/embryos in the oviduct environment. Maltose and glucose-1-phospate, energy substrates in oEVs, were by far the metabolites most affected by the cycle stage, with 8- to 100-fold higher levels at the luteal phase than at the pre- and post-ovulatory stages. Most pathways identified in the quantitative enrichment analysis including starch and sucrose metabolism, nucleotide sugars metabolism, glycolysis, lactose synthesis, and neoglucogenesis involved one or both of these carbohydrates. The sugar D-maltose is a glucoside consisting of two glucose monomers connected by an α-1,4-glycosidic bond. Maltose is usually the product of starch digestion, after alpha-amylase enzymatic action [35]. Although starch is not present in the oviduct, a large amount of glycogen was reported in the mammalian oviduct, where it acts as a glucose mobilized reservoir, storing up to 55,000 glucose moieties per molecule [36]. In the bovine oviduct epithelium, glycogen was quantified at around 60 µg/mg protein [37]. Most of the glucose residues in glycogen macromolecules are linked by α-1,4-glycosidic bonds while about one tenth of glucose residues are linked by α-1,6-glycosidic bonds. The enzymes alpha-amylase (AMY2B) and glycogen phosphorylase (PYGL, liver form and PYGB, brain form), all able to cut α-1,4-linkages, are produced in oviduct epithelial cells in mammals [30,36] and were reported at high abundance in the bovine OF [18,38]. Thus, it is likely that maltose and glucose-1-phosphate are products of glycogen catabolism in the oviduct epithelium. The abundance of maltose and glucose-1-phosphate in oEVs was upregulated during the luteal phase and reached its highest levels at the Late-lut stage. Similarly, glucose-1-phosphate in the bovine OF was reported to be 6-fold more abundant during the luteal phase than at the peri-ovulatory stages [15]. It is of note that levels of maltose and glucose-1-phosphate in oEVs paralleled those of P4 and were in opposition with those of E2 as previously reported in the bovine OF [19] and circulating plasma [39]. The hormonal regulation of glycogen metabolism in the oviduct remains unclear, but reproductive cycle-dependent changes in oviductal glycogen have been evidenced [36]. In pigs and rabbits, reserves of glycogen in the oviducts were highest near ovulation and decreased during the following luteal phase [40,41]. Furthermore, the hormonal control of endometrial glycogen has been well studied in rats, rabbits, and mink, where P4 promotes glycogen catabolism while E2 stimulates uterine glycogen synthesis and storage [36,42,43]. Altogether, these data suggest that the products of glycogen catabolism in the oviduct epithelium are abundantly released in oEVs under P4 action during the luteal phase and that this process is inhibited by E2 during estrus. The low levels of maltose and glucose-1-phosphate in oEVs during the peri-ovulatory period may play important roles for the success of reproduction. Interestingly, maltose has been shown to inhibit the binding of bovine and porcine spermatozoa to oviduct epithelial cell carbohydrates, a process allowing sperm storage and the delivery of capacitated spermatozoa to the site of fertilization [44,45]. The low levels of sources of glucose in oEVs in the post-ovulatory period are also concordant with the fact that exposure of the early bovine embryo to high amounts of glucose in vitro disturb embryo development and metabolism [46,47].

Additionally, we found that methionine concentrations in oEVs were higher at Pre-ov and Post-ov than at Late-lut. Methionine is a precursor for polypeptide synthesis and is an important factor in the antioxidant balance [48]. Furthermore, due to its role as the immediate precursor of S-adenosyl methionine, which is the universal methyl donor for epigenetic methylation of DNA and histones, methionine can influence the epigenetic programming of the embryo [49]. There is evidence that preimplantation embryo development is vulnerable to disruptions in methionine metabolism. For instance, disruption of methionine metabolism by ethionine, an antimetabolite of methionine, impairs the morula-to-blastocyst transition during bovine preimplantation development in vitro [50]. Supplementation of an in vitro embryo culture medium with large doses of S-adenosyl methionine was reported to cause a shift in bovine embryo sex ratio in favor of males and to induce a genome-wide hypermethylation [51].

Finally, we would like to discuss the unexpected presence of acetone, although at very low levels at all stages examined (≤1 nmol/mg of oEV protein), in oEVs. In dairy cows with subclinical or clinical ketosis, acetone concentrations in the circulating blood may reach 300–350 µmol/L [52,53]. Ketosis (or acetonemia) is a common disease in high-producing dairy cows caused by a negative energy balance and occurring mostly within two months after calving. Concentrations of acetone, acetoacetate, and β-hydroxybutyrate, three ketone bodies, increase in in blood and milk during ketosis [52,53]. This metabolic picture is typically associated with anestrus and lower fertility in dairy cows [54]. The ketosis status of the cows used for the present study could not been assessed at the time of oviduct collection, but they were mostly Holstein dairy cows, commonly regarded as particularly subject to ketosis. Thus, it is likely that the low concentrations of acetone detected in oEVs reflected low to moderate levels of acetone in the blood of animals at the time of slaughtering. However, why acetone levels in oEVs were lower at Pre-ov than at other stages of the estrous cycle is difficult to explain, with no data available in the current literature to shed some light.

In conclusion, our study provides the first comprehensive metabolomic profile of oEVs. Moreover, our results show that the hormonal environment of the estrus cycle affects the oEV-metabolite composition, with significant differences between the peri-ovulatory stages and late-luteal phase for specific metabolites. Our findings bring new insights into the roles of oEVs in supporting sperm function, fertilization, and early embryonic development.

## 4. Materials and Methods

### 4.1. Collection of Oviductal Fluids

Bovine oviducts were collected at a local slaughterhouse, transported on ice, and classified into four stages of the estrous cycle based on the ovarian and corpus luteum morphologies, as previously described [19]: post-ovulatory (Post-ov; Days 1–4 of estrous cycle, coinciding with the time of embryo presence in the oviduct), mid luteal (Mid-lut; Days 5–11), late luteal (Late-lut; Days 11–17), and pre-ovulatory (Pre-ov; Days 18–20, coinciding, if inseminated, with the time of sperm capacitation in the oviduct) (*n* = 34–54 cows/stage). The OF and epithelial cells were collected from the ipsilateral (to corpus luteum or Pre-ov follicle) and contralateral oviducts by gentle squeezing with a glass slide. Mixtures were kept on ice until OF was separated from cells by centrifugation at 2000× *g* for 15 min. Then, OF was stored at −80 °C until oEV isolation. To avoid the inclusion of cows with cystic follicles in the Pre-ov group, animals with a Pre-ov follicle larger than 20 mm in diameter were excluded at the time of oviduct collection. Furthermore, follicular fluid was collected from pre-ovulatory follicles and assayed for P4 and E2 concentrations by a competitive enzyme-linked immunosorbent assay [55] and the BioSource E2-EASIA Kit (BioSource, Louvain-la-Neuve, Belgium), respectively. Cows with intra-follicular concentrations of P4 higher than 160 ng/mL and of E2 lower than 40 ng/mL were also excluded, as previously described [19].

### 4.2. Isolation and Characterization of oEVs

#### 4.2.1. Preparation of Pools Oviductal Fluids and Isolation of oEVs

Preliminary experiments showed that 280 µL of OF was the minimal volume required for the detection of metabolites in oEVs by NMR. In order to reach at least three samples per estrous cycle stage and side, and based on the average volume of 30 µL of OF collected per oviduct, a minimum of 34 animals per stage were used (Table 1). To isolate oEVs, OF samples were thawed on ice and 3–4 biological pools were prepared by mixing OF from individual OF samples to reach a final minimum volume of 280 µL/pool (Table 1). Pools of OF were centrifuged at 12,000× *g* for 15 min to remove cellular debris and then ultracentrifuged twice at 100,000 g for 90 min to pellet the oEVs, as previously described [5]. The oEVs pellets were resuspended in 50 µL PBS, assayed for protein concentration by BCA (Bicinchoninic Acid) Protein Assay (ThermoFisher, Nanterre, France) and stored at −80 °C for further analysis.

#### 4.2.2. Oviductal EVs Observation by TEM

Aliquots of oEVs samples (one per stage and per side) were fixed with glutaraldehyde 2% for observation under TEM, as previously described [9]. The micrographs were obtained using TEM HITACHI HT 7700 Elexience at 80 kV (with a charge-coupled device camera AMT) and JEM 1011 (JEOL, Tokyo, Japan) equipped with a Gatan digital camera driven by Digital Micrograph software (Gatan, Pleasanton, CA, USA) at 100 kV. The processing of the photos and vesicle size calculation (min 300 vesicles per stage) were carried out by ImageJ software.

#### 4.2.3. Oviductal EVs Characterization by Western Blotting

Proteins (30 µg) were separated by SDS-PAGE and transferred onto nitrocellulose membranes (Whatman™, GE Healthcare Life Sciences, Amersham, UK). The membranes were blocked with Tris-buffered saline (TBS) containing Tween 20 (0.5% (*w/v*)) and supplemented with lyophilized low-fat milk (5% w/v) for 3 h at room temperature (TBS-T). The membranes were incubated with primary antibodies diluted in TBS-T containing 5% milk at 4 °C with gentle shaking overnight. The primary antibodies used were directed against CD81 (1:200 dilution; sc-166029, Santa Cruz Biotechnologies, Heidelberg, Germany), heat shock protein 70 (HSP70, 1:1000; Enzo, ADI-SPA-810), and annexin A1 (ANXA1, 1:400; Santa Cruz Biotechnologies, sc-114387). Subsequently, the membranes were washed with TBS-T and incubated at room temperature under agitation with secondary antibodies. The secondary antibodies used were Goat anti Mouse-HRP (1:5000; A4416, Merck, Saint-Quentin-Fallavier, France) for CD81 and HSP70 and Goat anti Rabbit-HRP (1:5000; A6154, Merck) for ANXA1. Blots were developed using a mixture of two chemiluminescence substrate developing kit (GE Healthcare AmershamTH ECL SelectTH Western blotting detection Reagent RPN2235 and Supersignal West Pico #34087 Chemiluminescent Substrate Thermo Scientific).

### 4.3. Proton Nuclear Magnetic Resonance Spectroscopy (1H-NMR) Analyses

Samples of oEVs were analyzed by NMR as previously described for bovine OF samples [15]. Briefly, 24 µL of oEV samples were diluted in 172 µL of 0.2 M potassium phosphate buffer in deuterium oxide (D2O) (pH 7.4 ± 0.5) and 4 µL of 3-(trimethylsilyl) propionic-acid solution at 3.2 mM, used as a spectrometer field lock signal. The resulting solution was transferred to conventional 3-mm NMR tubes. Briefly, 1H-NMR spectra were recorded at 298 K on a Bruker Ascend 600 MHz spectrometer (Bruker, Sadis, Wissembourg, France), equipped with a TCI cryoprobe. Standard NMR spectra were obtained using a “cpmgpr1d” pulse sequence with a 90° pulse, a relaxation delay of 25 s, number of scans of 256 on a time domain of 64 K data points. Data were processed with 0.2 Hz of line broadening for the exponential decay function using TopSpin version 3.2 software (Bruker daltonik, Karlshure, Germany). Metabolite quantifications using the ERETIC peak as a quantitative reference were obtained by the specific subroutine of the Bruker TopSpin 3.2 program. The ERETIC signal was calibrated on a known concentration amino-acid solution ([ERETIC] = 198 μM). Spectral assignments were done using the free version of ChenomX 7.1 software (ChenomX, Edmonton, Canada), in house database, and human metabolome database (HMDB version 4.0; http://www.hmdb.ca, assessed 18 March 2019) [56]. When the assignment was uncertain, spiking experiments were used to validate metabolite assignments. Comparing the NMR spectra obtained from a biological sample spiked-in with a known metabolite with that obtained without spiking made it possible to confirm or discard the presence or absence of this metabolite in the biological sample.

### 4.4. Data Processing and Statistical Analysis of NMR Data

First, the concentration of each metabolite was normalized to 1 mg of oEV protein. Then, to obtain an overview of the oEV metabolite levels across the different stages of the estrous cycle, principal component analysis (PCA) was generated using normalized metabolite data in RStudio (R packages: ggplot2, ggfortify). Statistical analyses were performed using the GraphPad Prism software (version 7, San Diego, CA, USA). Normalized values were compared between stages of the cycle and sides relative to ovulation with a two-way analysis of variance (ANOVA) followed by Tukey post-tests. When no side × stage and side effect (*p* > 0.05) were identified, data for ipsilateral and contralateral oviducts were combined and an average value calculated for each stage. A *p*-value < 0.05 was considered as significant. Data are presented as means ± SEM.

### 4.5. Pathway Enrichment Analyses of NMR Data

To investigate the presence of biologically meaningful patterns among the oEV metabolites, metabolic pathway analysis was performed using the functional enrichment tool of the free web-based software MetaboAnalyst 4.0 (https://www.metaboanalyst.ca/; assessed 12–24 June 2019) [57]. First, enrichment over representation analysis (ORA) was performed using the list of all metabolites identified in oEVs. Then, to obtain further insights on the regulated metabolic pathways across the cycle, a quantitative enrichment analysis by paired comparisons between stages using normalized (log transformed) concentrations of quantified metabolites was performed. The *p*-value and the false discovery rate (FDR) of each regulated pathway were used to account for significance and false discovery in the pathway analysis, respectively. In this regard, the thresholds of *p*-value < 0.05 and FDR < 0.1 were considered as significant.

## Figures and Tables

**Table 1:** Samples of bovine oviductal extracellular vesicles (oEVs) used for proton nuclear magnetic resonance spectroscopy (NMR) analysis.

Stage of the Cycle	No. Animals ^1^	Side Relative to Ovulation	Number of OF Pools	Volume (µL) of OF before oEV Isolation	Protein Concentration (mg mL^−1^)
Post-ovulatory	54	Ipsilateral	4	320–420	10.4–13.6
		Contralateral	4	288–385	9.7–17.4
Mid-luteal	40	Ipsilateral	3	355–400	11.4–12.6
		Contralateral	3	305–410	11.1–13.5
Late-luteal	34	Ipsilateral	3	315–470	9.4–10.3
		Contralateral	3	350–470	13.1–15.9
Pre-ovulatory	40	Ipsilateral	3	450–495	10.0–15.2
		Contralateral	3	500–580	12.0–13.4

^1^ Number of animals used to obtain 3–4 pools of oviductal fluid (OF) per stage × side with a minimum volume of 280 µL/pool that was used for oEV isolation.

**Table 2:** Metabolites identified in bovine oviductal extracellular vesicles across the estrous cycle, ordered by chemical classes ^1^.

Metabolite	Chemical Taxonomy Subclass Description	Identified (I)/Quantified (Q)	Function
Valine	Amino acids, peptides, and analogues (Proteinogenic) Essential amino acids	I/Q	Involved in stress, energy and muscle metabolism
Threonine	Amino acids, peptides, and analogues (Proteinogenic) Essential amino acids	I	Involved in biosynthesis of proteins
Methionine	Amino acids, peptides, and analogues (Proteinogenic) Essential amino acids	I/Q	Required for normal growth and development
Leucine	Amino acids, peptides, and analogues (Proteinogenic) Essential amino acids	I	Involved in biosynthesis of proteins, stress, energy, and muscle metabolism. Stimulates insulin release
Isoleucine	Amino acids, peptides, and analogues (Proteinogenic) Essential amino acids	I/Q	Involved in stress, energy and muscle metabolism.
Proline	Amino acids, peptides, and analogues Conditionally essential amino acids (Proteinogenic)	I	Involved in biosynthesis of proteins
Glycine	Amino acids, peptides, and analogues Non-essential amino acids (Proteinogenic)	I/Q	Involved in the body’s production of DNA, phospholipids, and collagen, and in release of energy.
Alanine	Amino acids, peptides, and analogues Non-essential amino acids (Proteinogenic)	I/Q	One of the most important amino acids released by muscle, functioning as a major energy source. Regulator of glucose metabolism, lymphocyte reproduction and immunity.
Glutamate	Amino acids, peptides, and analogues (Non-essential alpha Amino Acids)	I	Involved in biosynthesis of proteins Role as neurotransmitter, a chemical used by nerve cells to send signals to other cells.
Creatine	Amino acids, peptides, and analogues (endogenous amino acid: synthesized from arginine, glycine, and methionine)	I/Q	Role in energy metabolism. Responsible for the production of ATP in skeletal muscle through the process of oxidative phosphorylation inside the mitochondria.
Ethanolamine	Amines	I	Widely distributed in biological tissue and is a component of lecithin.
Carnitine	Quaternary ammonium salts Non-proteinogenic amino acids	I/Q	Important in providing energy to muscles Described as a vitamin, an amino acid, or essential metabolite
Choline	Quaternary ammonium salts (Essential Vitamin)	I/Q	Considered an essential vitamin. Precursor for the neurotransmitter acetylcholine, which is involved in many functions including memory and muscle control.
Lactate	Alpha hydroxy acids and derivatives (Energy Substrate)	I/Q	Plays a role in several biochemical processes and is produced in the muscles during intense activity
Myoinositol	Alcohols and polyols	I/Q	Involved in the Inositol phosphate metabolism and the Phosphatidylinositol signaling system.
Glucose-1-phosphate	Carbohydrates and carbohydrate conjugates (Energy Substrate)	I/Q	Glycogenolysis produces glucose-1-phosphate and no energy.
Maltose	Carbohydrates and carbohydrate conjugates (Energy Substrate)	I/Q	Maltose can be broken down into two glucose molecules
Glucose	Carbohydrates and carbohydrate conjugates (Energy Substrate)	I	Primary source of energy
Succinate	Dicarboxylic acids and derivatives	I/Q	Component of the citric acid or TCA cycle and is capable of donating electrons to the electron transfer chain.
Formate	Carboxylic acids	I	Essential intermediary metabolite in folate-mediated one-carbon metabolism Responsible for both metabolic acidosis and disrupting mitochondrial electron transport and energy production.
Acetate	Carboxylic acid derivates	I/Q	Acetate in the form of acetyl CoA is used in metabolism to yield chemical energy.
Acetone	Carbonyl compounds	I/Q	One of the ketone bodies produced during ketoacidosis. Since ketosis develops under serious metabolic circumstances, all the mechanisms that balance or moderate the effects of ketosis enhance the chance for survival.

^1^ Information detailed on the table was obtained from “The human metabolome database” (http://www.hmdb.ca/).

**Table 3:** Concentrations of metabolites in bovine oviductal extracellular vesicles according to the stage of the estrous cycle and the side relative to ovulation (ipsilateral vs. contralateral). Data are given as means ± SEM of nmoL mg^−1^ of oEV proteins.

	Pre-Ovulatory		Post-Ovulatory		Mid-Luteal		Late-Luteal	
	Ipsi	Contra	Ipsi	Contra	Ipsi	Contra	Ipsi	Contra
Acetate	2.5 ± 0.6	2.8 ± 0.1	2.7 ± 0.2	2.7 ± 0.2	2.9 ± 0.3	2.8 ± 0.0	2.8 ± 0.2	2.8 ± 0.2
Acetone	0.6 ± 0.2	0.7 ± 0.0	0.9 ± 0.0	0.9 ± 0.0	1.0 ± 0.1	0.9 ± 0.0	1.0 ± 0.1	0.9 ± 0.0
Alanine	16.7 ± 3.7	18.1 ± 0.9	15.5 ± 1.6	14.9 ± 1.4	15.5 ± 2.3	13.8 ± 0.3	13.4 ± 0.9	13.8 ± 1.6
Carnitine	4.8 ± 0.8	6.1 ± 0.2	4.3 ± 0.4	4.9 ± 0.6	5.5 ± 0.6	5.7 ± 0.9	5.5 ± 0.8	5.6 ± 0.8
Choline	8.8 ± 1.6	9.4 ± 0.5	10.9 ± 0.9	9.7 ± 0.8	10.3 ± 1.7	10.2 ± 0.4	8.6 ± 0.4	9.5 ± 1.2
Creatine	7.8 ± 1.8	8.4 ± 0.4	7.7 ± 0.6	7.8 ± 0.6	7.9 ± 0.9	7.9 ± 0.5	7.7 ± 0.6	7.7 ± 0.9
Glucose-1-phosphate	1.1 ± 0.6	0.7 ± 0.5	0.8 ± 0.5	3.1 ± 1.5	9.8 ± 2.1	39.5 ± 5.7	87.2 ± 41.8	117.1 ± 1.4
Glycine	25.2 ± 5.0	31.4 ± 5.7	30.8 ± 3.1	25.0 ± 2.1	39.3 ± 6.4	26.5 ± 0.9	27.5 ± 1.8	25.8 ± 4.2
Isoleucine	2.6 ± 0.5	3.2 ± 0.2	2.8 ± 0.3	2.6 ± 0.3	2.7 ± 0.3	2.5 ± 0.0	2.5 ± 0.4	2.4 ± 0.4
Lactate	33.0 ± 6.4	37.7 ± 2.4	36.7 ± 3.1	36.9 ± 2.5	43.0 ± 4.1	41.9 ± 2.7	43.3 ± 6.9	43.7 ± 6.1
Maltose	13.9 ± 5.0	19.1 ± 5.0	14.1 ± 1.7	29.3 ± 10.6	53.2 ± 4.3	110.5 ± 13.1	203.7 ± 9.7	225.1 ± 32.9
Methionine	10.0 ± 1.9	9.8 ± 0.6	8.7 ± 1.0	9.2 ± 1.0	7.3 ± 0.9	7.4 ± 0.6	6.2 ± 0.8	6.4 ± 0.8
Myoinositol	37.6 ± 7.9	41.5 ± 2.2	43.8 ± 4.2	44.4 ± 4.2	50.9 ± 5.5	51.5 ± 3.1	47.8 ± 5.7	50.4 ± 6.2
Succinate	0.7 ± 0.2	0.7 ± 0.0	0.9 ± 0.1	0.8 ± 0.1	1.1 ± 0.1	0.9 ± 0.1	1.0 ± 0.2	0.8 ± 0.2
Valine	3.2 ± 0.8	3.8 ± 0.2	3.3 ± 0.4	3.3 ± 0.4	3.2 ± 0.5	2.9 ± 0.0	2.7 ± 0.2	2.7 ± 0.2

**Table 4:** Over representation analysis of all identified metabolites in oEVs against pathway associated metabolite sets. The associated bar chart visualization of this analysis is shown in Figure 4.

Pathway Associated Metabolite Sets ^1^	Implicated Metabolites ^2^	Total ^3^	Hits ^4^	Raw p ^5^	Holm p ^6^	FDR ^7^
Glycine and serine metabolism	Creatine, Glycine, L-glutamic acid, L-alanine, L-threonine, L-methionine	59	6	7.82 × 10^−4^	0.0766	0.0766
Arginine and Proline Metabolism	Creatine, Glycine, L-glutamic acid, L-proline, Succinic acid	53	5	0.00332	0.322	0.137
Alanine metabolism	Glycine, L-glutamic acid, L-alanine	17	3	0.00421	0.404	0.137
Valine, Leucine and Isoleucine degradation	L-glutamic acid, L-isoleucine, Succinic acid, L-leucine, L-valine	60	5	0.00575	0.546	0.141
Glutathione Metabolism	Glycine, L-glutamic acid, L-alanine	21	3	0.0078	0.734	0.146
Carnitine Synthesis	L-carnitine, Glycine, Succinic acid	22	3	0.00892	0.829	0.146
Glutamate Metabolism	Glycine, L-glutamic acid, L-alanine, Succinic acid	49	4	0.0152	1	0.212
Phosphatidylethanolamine biosynthesis	Choline, Ethanolamine	12	2	0.0234	1	0.267
Ketone Body Metabolism	Succinic acid, Acetone	13	2	0.0273	1	0.267
Glucose-Alanine Cycle	L-glutamic acid, L-alanine	13	2	0.0273	1	0.267
Phosphatidylcholine biosynthesis	Choline, Ethanolamine	14	2	0.0314	1	0.28

^1^ Only pathway associated metabolite sets with *p* < 0.05 are shown in the table; ^2^ Implicated metabolites from oEVs in the associated metabolite set; ^3^ Total number of metabolites in the metabolite set; ^4^ Hits: number of metabolites from oEVs involved in the metabolite set; ^5^ Raw *p*: original *p* value calculated from the enrichment analysis; ^6^ Holm *p*: adjusted raw *p* value by the Holm–Bonferroni method; ^7^ FDR: false discovery rate.

**Table 5:** Quantitative enrichment analysis of bovine oEV metabolites against pathway associated metabolite sets. Associated bar chart visualization of this analysis is shown in Figure 5.

Pathway Associated Metabolite Sets ^1^	Implicated Metabolites ^2^	Total ^3^	Hits ^4^	Raw p ^5^	Holm p ^6^	FDR ^7^
Starch and sucrose metabolism	D-maltose, Glucose-1-phosphate	31	2	3.21 × 10^−5^	0.0015	6.835 × 10^−4^
Nucleotide sugars metabolism	Glucose-1-phosphate	20	1	8.651 × 10^−5^	0.0039	6.835 × 10^−4^
Glycolysis	Glucose-1-phosphate	25	1	8.651 × 10^−5^	0.0039	6.835 × 10^−4^
Lactose synthesis	Glucose-1-phosphate	20	1	8.651 × 10^−5^	0.0039	6.835 × 10^−4^
Gluconeogenesis	Glucose-1-phosphate, L-lactic acid	35	2	8.662 × 10^−5^	0.0039	6.835 × 10^−4^
Galactose metabolism	Myoinositol, Glucose-1-phosphate	38	2	8.726 × 10^−5^	0.1933	6.835 × 10^−4^
Spermidine and spermine biosynthesis	L-methionine	18	1	0.0047	0.4906	0.032
Betaine metabolism	Choline, L-methionine	21	2	0.0123	1	0.072
Methionine metabolism	Choline, Glycine, L-methionine	43	3	0.0438	1	1

^1^ Only pathway associated metabolite sets with *p* < 0.05 are shown in the table; ^2^ Implicated metabolites from oEV in the associated metabolite set; ^3^ Total number of metabolites in the metabolite set; ^4^ Hits: number of metabolites from oEV involved in the metabolite set; ^5^ Raw *p*: original *p* value calculated from the enrichment analysis; ^6^ Holm *p*: adjusted raw *p* value by the Holm–Bonferroni method; ^7^ FDR: false discovery rate.

## Abbreviations

NMR	Nuclear magnetic resonance
oEVs	Oviductal extracellular vesicles
OF	Oviductal fluid
